# Supplementary material for: Photoactivatable ribonucleosides mark base-specific RNA-binding sites
Source: Nat Commun. 2021 Oct 15;12:6026. doi: 10.1038/s41467-021-26317-5 (PMC8519950; doi:10.1038/s41467-021-26317-5)
Supplement: Supplementary file 1 — Supplementary Information [file 41467_2021_26317_MOESM1_ESM.pdf]

Supplementary Information

Photoactivatable ribonucleosides mark base-specific RNA-binding sites

Jong Woo Bae[1,2], Sangtae Kim[3], V. Narry Kim[1,2]*, and Jong-Seo Kim[1,2]*
*Correspondence: jongseokim@snu.ac.kr and narrykim@snu.ac.kr

1 Center for RNA Research, Institute for Basic Science, Seoul 08826, Korea
2 School of Biological Sciences, Seoul National University, Seoul 08826, Korea
3 Seer Inc., Redwood City, CA, 94065, USA

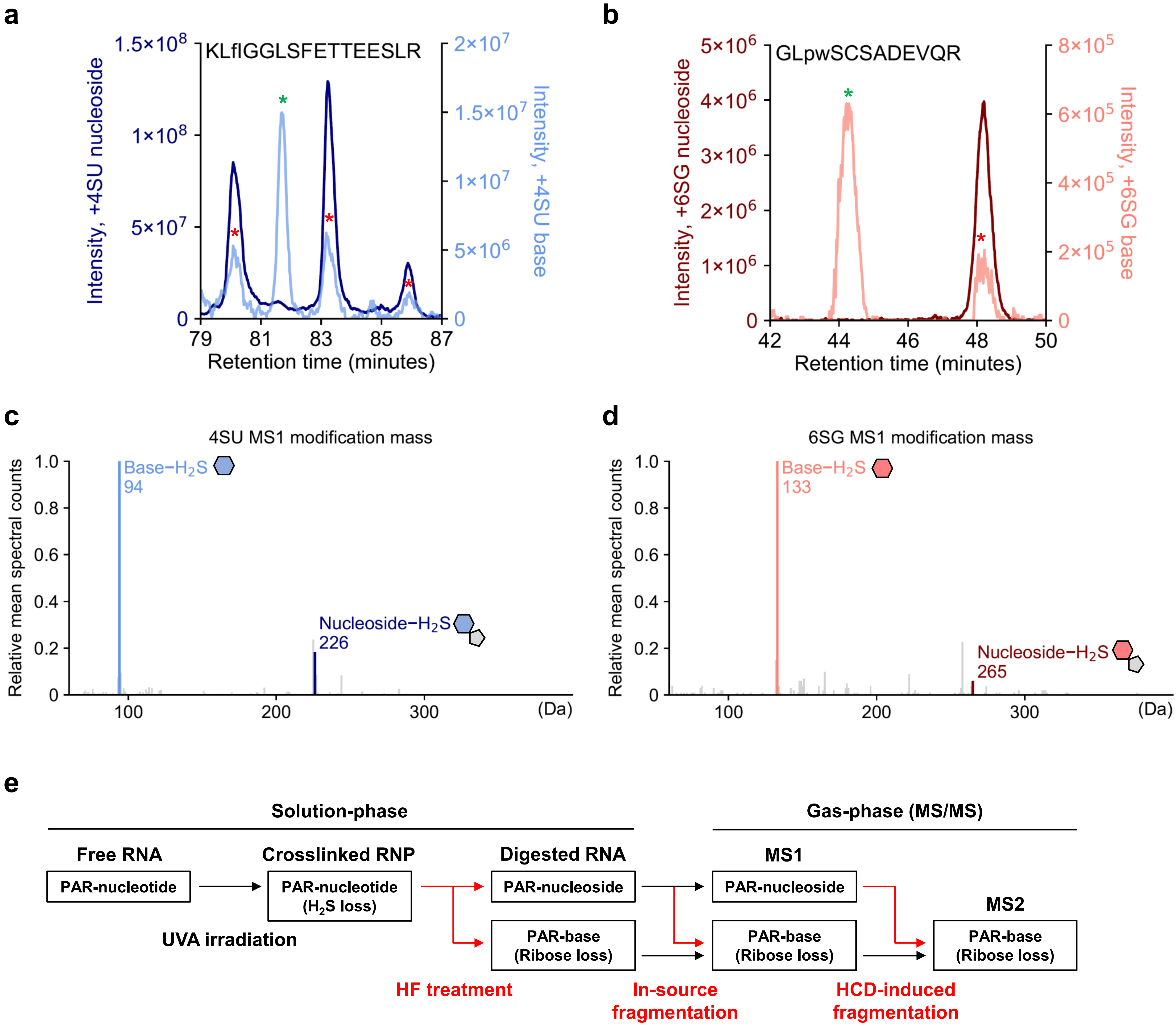

Supplementary Figure 1. Liquid chromatographic and mass spectrometric features of PAR-RBSs

a Coelution pattern and relative abundance of extracted ion chromatograms (XICs) of precursor ions corresponding to the triply charged peptide KLFIGGLSFETTEESLR with 4SU-nucleoside (m/z range: 718.34-718.38 Th) or base (m/z range: 674.33-674.37 Th) modification. For the latter, XIC peaks in the same retention time as the former (products of gas-phase in-source fragmentation during MS/MS) or not (products of in-solution HF digestion) were labeled with green or red stars, respectively. Amino acids identified as the best localization site at least once in MSFragger open search were depicted in lowercase. **b** Same analysis as in **a** on the doubly charged peptide GLPWSC(+carbamidomethylation)SADEVQR with 6SG-nucleoside (m/z range: 885.37-885.41 Th) or base (m/z range: 819.35-819.39 Th) modification. **c-d** Open search for modified mass on 4SU-RBS (**c**) or 6SG-RBS (**d**) using MODa. Modification mass ranges of 60-400 Da are shown. **e** Chemical features and fragmentation patterns of PAR adducts both in solution and gas phases.

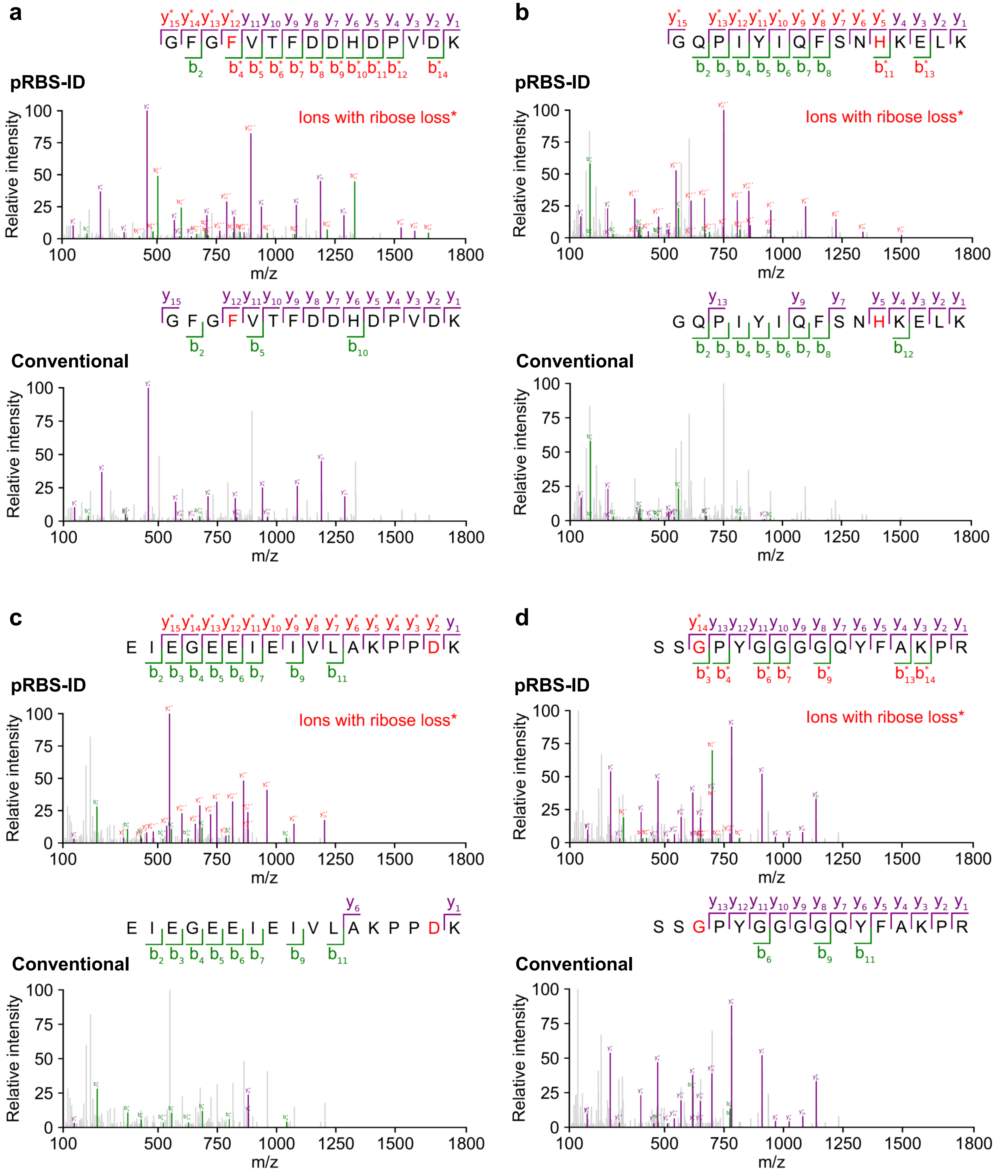

Supplementary Figure 2. Representative MS2 spectra of 4SU-nucleoside-crosslinked peptides

a-d Annotated MS2 spectra of peptides with aromatic (Phe) (**a**), basic (His) (**b**), acidic (Asp) (**c**), small (Gly) (**d**) amino acids crosslinked to 4SU-nucleoside. Fragment ions assuming ribose neutral loss (top, pRBS-ID) or intact modification (bottom, Conventional) were annotated separately.

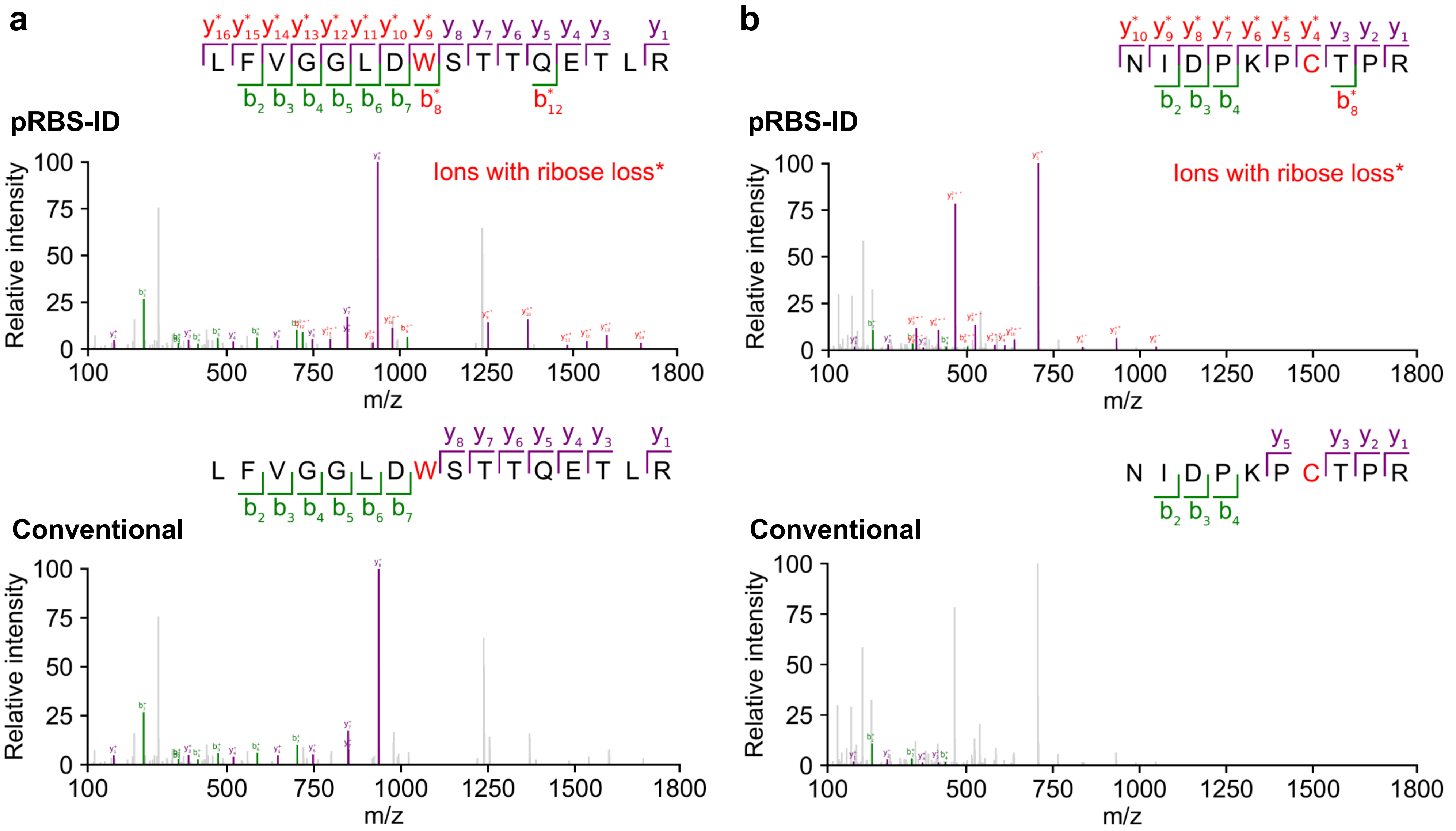

Supplementary Figure 3. Representative MS2 spectra of 6SG-nucleoside-crosslinked peptides

a-b Annotated MS2 spectra of peptides with aromatic (Trp) (**a**) or nucleophilic (Cys) (**b**) amino acids crosslinked to 6SG-nucleoside. Fragment ions assuming ribose neutral loss (top, pRBS-ID) or intact modification (bottom, Conventional) were annotated separately.

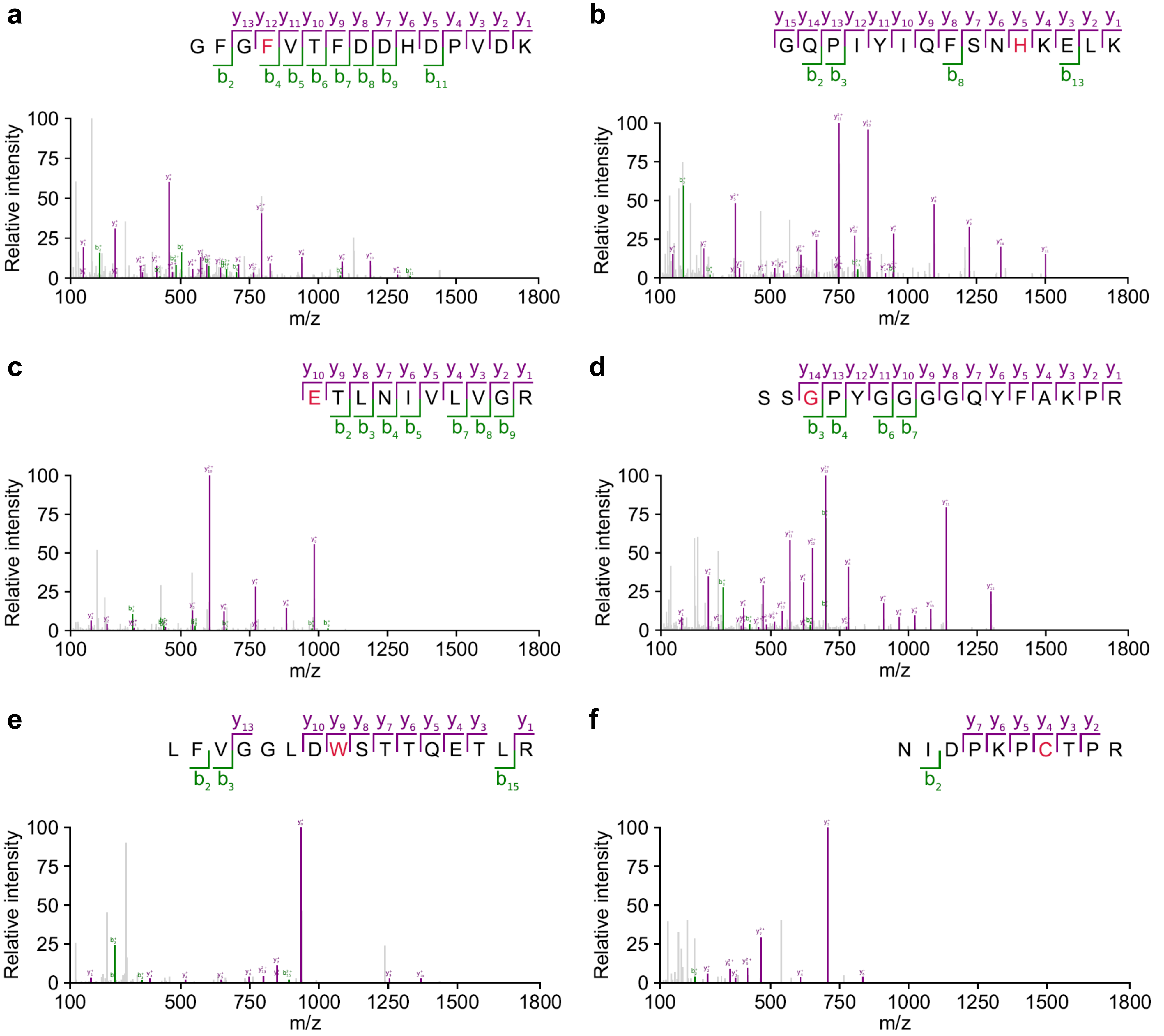

Supplementary Figure 4. Representative MS2 spectra of PAR-base-crosslinked peptides

a-d Annotated MS2 spectra of peptides with aromatic (Phe) (**a**), basic (His) (**b**), acidic (Glu) (**c**), small (Gly) (**d**) amino acids crosslinked to 4SU-base. **e-f** Annotated MS2 spectra of peptides with aromatic (Trp) (**e**) or nucleophilic (Cys) (**f**) amino acids crosslinked to 6SG-base.

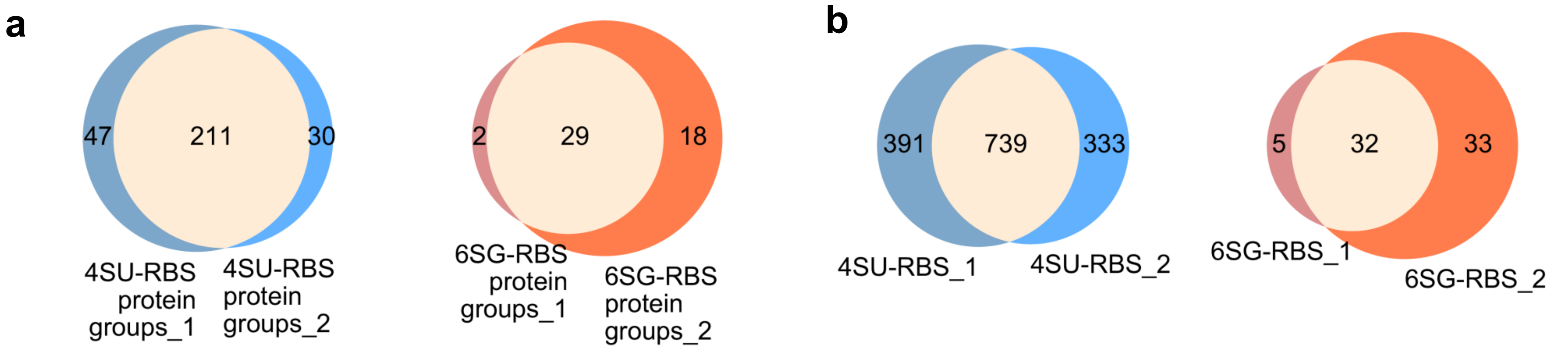

Supplementary Figure 5. RBS identification results from pRBS-ID experiments

a Inter-replicate comparison of identified protein groups with 4SU-RBSs (left) and 6SG-RBSs (right). **b** Inter-replicate comparison of identified 4SU-RBSs (left) and 6SG-RBSs (right).

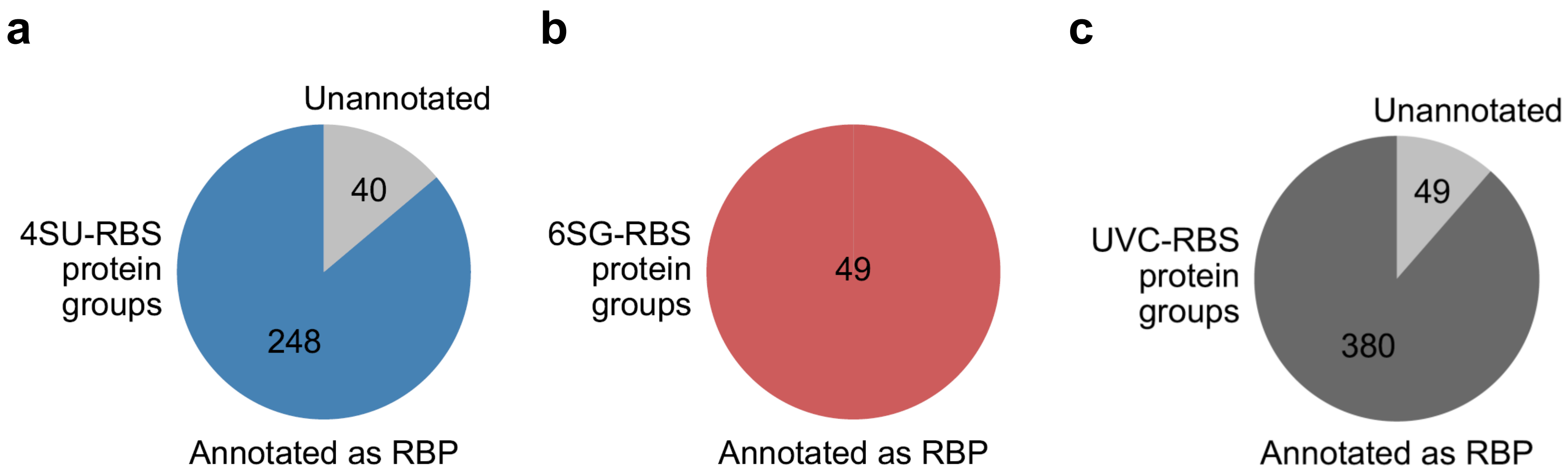

Supplementary Figure 6. RBP annotation in protein groups with RBSs identified
a-c Proportion of RBP annotation in protein groups containing 4SU-RBSs (**a**), 6SG-RBSs (**b**), or UVC-RBSs (**c**).

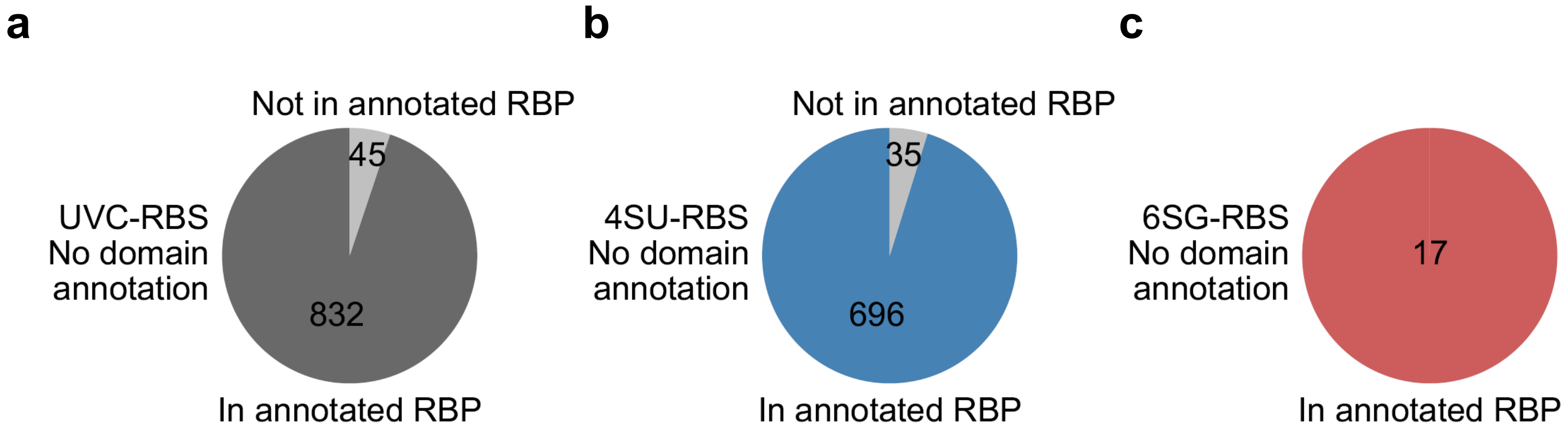

Supplementary Figure 7. RBSs identified in novel RNA-binding regions of RBPs

a-c RBP annotations of UVC-RBS (**a**), 4SU-RBS (**b**), or 6SG-RBS (**c**) identified in regions without domain annotation, respectively.

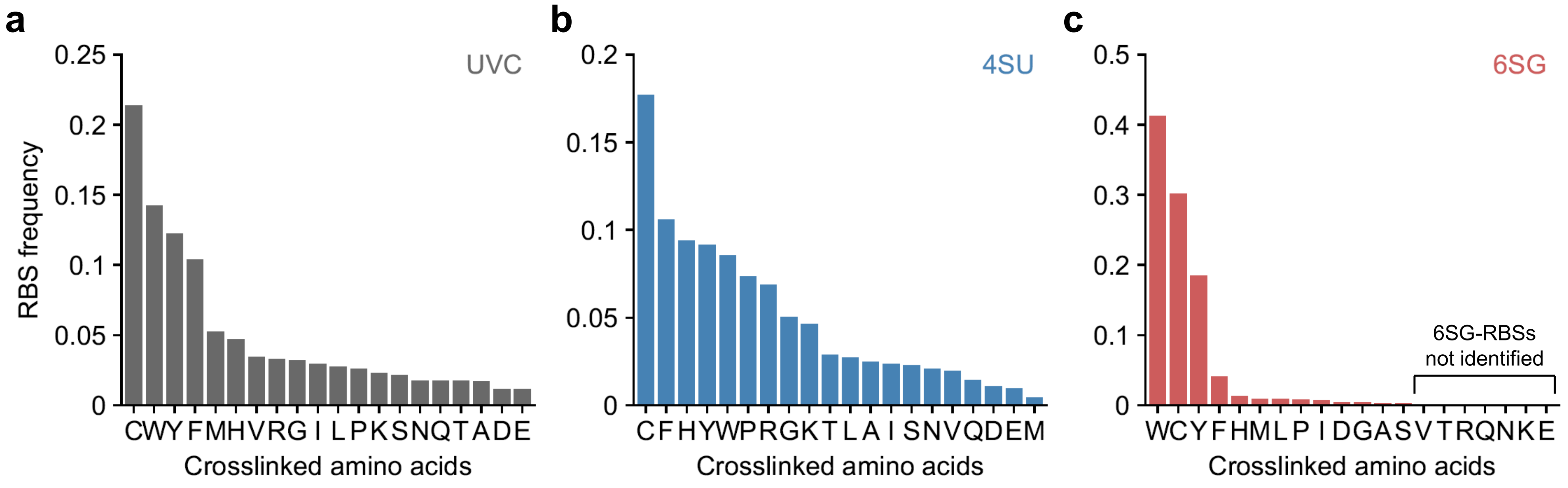

Supplementary Figure 8. Frequencies of 20 amino acids in RBS datasets

a-c Amino acid frequencies of UVC-RBS (**a**), 4SU-RBS (**b**), or 6SG-RBS (**c**), respectively normalized by those of all sequences in proteins where each RBS type was identified.

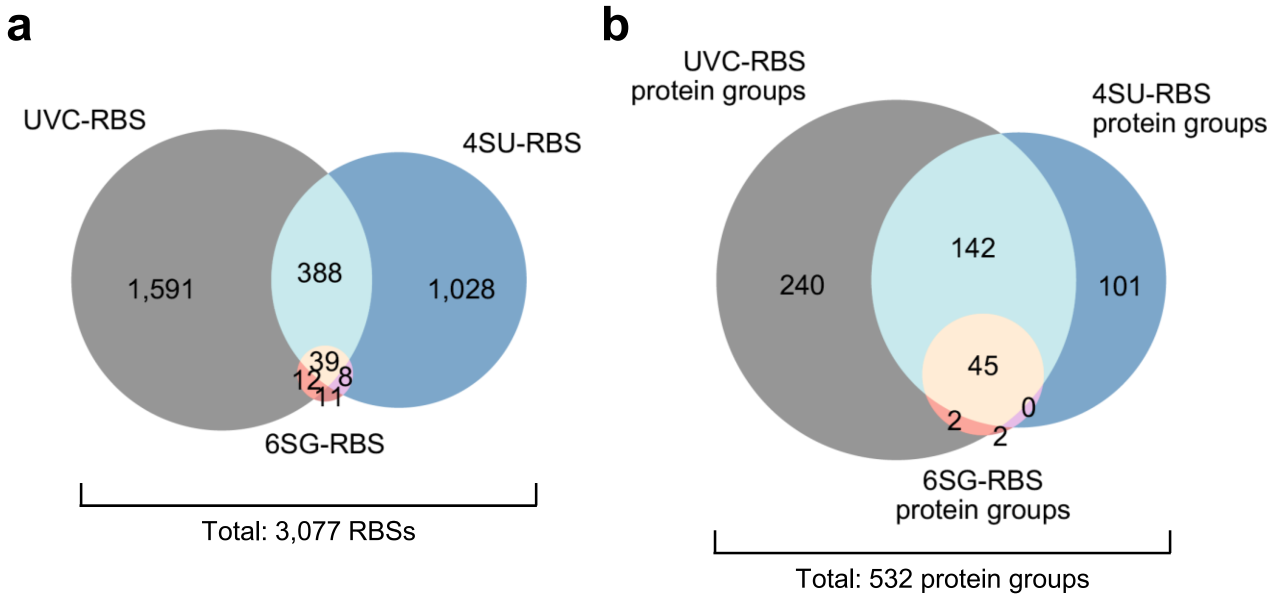

Supplementary Figure 9. Collective RBS identification results from RBS-ID and pRBS-ID experiments

a-b Identified UVC-RBSs, 4SU-RBSs, and 6SG-RBSs (**a**) and the respective protein groups (**b**).
