# Supplementary material for: Photoactivatable ribonucleosides mark base-specific RNA-binding sites
Source: Nat Commun. 2021 Oct 15;12:6026. doi: 10.1038/s41467-021-26317-5 (PMC8519950; doi:10.1038/s41467-021-26317-5)
Supplement: Supplementary file 3 — Description of Additional Supplementary Files [file 41467_2021_26317_MOESM3_ESM.pdf]

Description of Additional Supplementary Files

File Name: Supplementary Data 1
Description: 4SU- and 6SG-RBS open search results

File Name: Supplementary Data 2
Description: UVC-, 4SU- and 6SG-RBS closed search results
