# Supplementary material for: Photoactivatable ribonucleosides mark base-specific RNA-binding sites
Source: Nat Commun. 2021 Oct 15;12:6026. doi: 10.1038/s41467-021-26317-5 (PMC8519950; doi:10.1038/s41467-021-26317-5)
Supplement: Supplementary file 6 — Reporting Summary [file 41467_2021_26317_MOESM6_ESM.pdf]

nature research

Corresponding author(s): V. Narry Kim, Jong-Seo Kim

Last updated by author(s): Sep 27, 2021

Reporting Summary

Nature Research wishes to improve the reproducibility of the work that we publish. This form provides structure for consistency and transparency in reporting. For further information on Nature Research policies, see our Editorial Policies and the Editorial Policy Checklist.

Statistics

For all statistical analyses, confirm that the following items are present in the figure legend, table legend, main text, or Methods section.

n/a	Confirmed	
[ ]	[x]	The exact sample size (*n*) for each experimental group/condition, given as a discrete number and unit of measurement
[ ]	[x]	A statement on whether measurements were taken from distinct samples or whether the same sample was measured repeatedly
[x]	[ ]	The statistical test(s) used AND whether they are one- or two-sided<br>*Only common tests should be described solely by name; describe more complex techniques in the Methods section.*
[x]	[ ]	A description of all covariates tested
[x]	[ ]	A description of any assumptions or corrections, such as tests of normality and adjustment for multiple comparisons
[ ]	[x]	A full description of the statistical parameters including central tendency (e.g. means) or other basic estimates (e.g. regression coefficient) AND variation (e.g. standard deviation) or associated estimates of uncertainty (e.g. confidence intervals)
[x]	[ ]	For null hypothesis testing, the test statistic (e.g. *F*, *t*, *r*) with confidence intervals, effect sizes, degrees of freedom and *P* value noted<br>*Give P values as exact values whenever suitable.*
[x]	[ ]	For Bayesian analysis, information on the choice of priors and Markov chain Monte Carlo settings
[x]	[ ]	For hierarchical and complex designs, identification of the appropriate level for tests and full reporting of outcomes
[ ]	[x]	Estimates of effect sizes (e.g. Cohen's *d*, Pearson's *r*), indicating how they were calculated

Our web collection on statistics for biologists contains articles on many of the points above.

Software and code

Policy information about availability of computer code

Data collection	Thermo Scientific Xcalibur (v.3.1) was used to collect mass spectrometry data.
Data analysis	RawConverter (v.1.1.0.19), msconvert (v.3.0.1908), mPE-MMR (v.1.1.8), MSFragger (v.20190530), MODa (v1.6.0), and MS-GF+ (v.20170127) were used to analyze raw files in mass spectrometry experiments. PyMOL (v.1.7.2.1) was used to visualize previously published structural data. Python 3.7-based custom codes used to for post-processing of the analyzed data were described in detail in the online methods, as well as deposited in the GitHub repository (https://doi.org/10.5281/zenodo.5527793).

For manuscripts utilizing custom algorithms or software that are central to the research but not yet described in published literature, software must be made available to editors and reviewers. We strongly encourage code deposition in a community repository (e.g. GitHub). See the Nature Research guidelines for submitting code & software for further information.

Data

Policy information about availability of data

All manuscripts must include a data availability statement. This statement should provide the following information, where applicable:

- Accession codes, unique identifiers, or web links for publicly available datasets
- A list of figures that have associated raw data
- A description of any restrictions on data availability

The mass spectrometry proteomics data generated in this study have been deposited in the ProteomeXchange Consortium PRIDE database under accession code PXD023401. The structural data used in this study are available in the RCSB Protein Data Bank database under accession codes 2ADC, 2KFY, 4V6X, and 6ZVH. The raw data were used to generate Figs. 1, 2, 3, and 4. Source data are provided with this paper.

Field-specific reporting

Please select the one below that is the best fit for your research. If you are not sure, read the appropriate sections before making your selection.

- [x] Life sciences
- [ ] Behavioural & social sciences
- [ ] Ecological, evolutionary & environmental sciences

For a reference copy of the document with all sections, see nature.com/documents/nr-reporting-summary-flat.pdf

Life sciences study design

All studies must disclose on these points even when the disclosure is negative.

Sample size	All experimental data are available in biological duplicates. The sample size (n=2) was empirically chosen because it was sufficient to demonstrate the reproducibility of the biochemical mass spectrometry experiments. No statistical tests were used.
Data exclusions	No data were excluded from analysis.
Replication	For all experiments, all attempts at replication were successful in biological duplicates.
Randomization	This is irrelevant to our study. This is because our method-oriented study only used a single cell line, and did not require any comparison using multiple experimental groups.
Blinding	This is irrelevant to our study. This is because our method-oriented study only used a single cell line, and did not require any comparison using multiple experimental groups.

Reporting for specific materials, systems and methods

We require information from authors about some types of materials, experimental systems and methods used in many studies. Here, indicate whether each material, system or method listed is relevant to your study. If you are not sure if a list item applies to your research, read the appropriate section before selecting a response.

Materials & experimental systems

n/a	Involved in the study	
x		Antibodies
	x	Eukaryotic cell lines
x		Palaeontology and archaeology
x		Animals and other organisms
x		Human research participants
x		Clinical data
x		Dual use research of concern

Methods

n/a	Involved in the study	
x		ChIP-seq
x		Flow cytometry
x		MRI-based neuroimaging

Eukaryotic cell lines

Policy information about cell lines

Cell line source(s)	HeLa cells were generous gifts from laboratories in School of Biological Sciences, Seoul National University. Commercial sources are not available.
Authentication	HeLa cells were authenticated by ATCC, via STR Profiling following ISO 9001:2008 and ISO/IEC 17025:2005 quality standards.
Mycoplasma contamination	HeLa was tested negative for mycoplasma contamination.
Commonly misidentified lines (See ICLAC register)	No commonly misidentified cell lines were used.
